# Sexual Health

**DOI:** 10.1186/1472-6874-4-S1-S24

**Published:** 2004-08-25

**Authors:** Lisa Hansen, Janice Mann, Sharon McMahon, Thomas Wong

**Affiliations:** 1 Centre for Infectious Disease Prevention and Control, Health Canada, Tunney's Pasture, Ottawa, Canada; 2 Centre for Infectious Disease Prevention and Control, Health Canada, Tunney's Pasture, Ottawa, Canada; 3 Centre for Infectious Disease Prevention and Control, Health Canada, Tunney's Pasture, Ottawa, Canada; 4 Centre for Infectious Disease Prevention and Control, Health Canada, 400 Cooper Street, Suite 2005, Ottawa, Canada

## Abstract

**Health Issue:**

Much attention is devoted to women's reproductive health, but the formative and mature stages of women's sexual lives are often overlooked. We have analyzed cross-sectional data from the Sexual Behaviour module of the 2000/2001 Canadian Community Health Survey (CCHS), and reviewed the literature and available indicators of the sexual health of Canadian women.

**Key Findings:**

Contemporary Canadian adolescents are becoming sexually active at younger ages than in previous generations. The gender gap between young males and females in age at first intercourse has virtually disappeared. The mean age at first intercourse for CCHS respondents aged 15–24 years was between 16 and 17. Canadian-born respondents are significantly younger at first intercourse than those who were born outside of Canada. Few adolescents recognize important risks to their sexual health. Older Canadians are sexually active, and continue to find emotional and physical satisfaction in their sexual relationships.

**Data Gaps and Recommendations:**

Both health surveys and targeted research must employ a broader understanding of sexuality to measure changes in and determinants of the sexual health of Canadians. There is reluctance to direct questions about sexual issues to younger Canadians, even though increased knowledge of sexual health topics is associated with delayed onset of sexual intercourse. Among adults, sex-positive resources are needed to address aspects of aging, rather than medicalizing age-related sexual dysfunction. Age and gender-appropriate sexual health care, education, and knowledge are important not only for women of reproductive age, but for Canadians at all stages of life.

## Background

### What is Sexual Health?

Sexual health, far from being merely the absence of disease or dysfunction, is a vital and essential part of being human. The Pan American Health Organization and World Health Organization recently defined sexual health as "the experience of the ongoing process of physical, psychological and sociocultural well being related to sexuality."[1] Health Canada's 1999 *Report from Consultations on a Framework for Sexual and Reproductive Health *has asserted as a guiding principle that all individuals are sexual beings throughout their lives,[2] and this is the broad approach taken here in considering issues in Canadian women's sexual health.

### Sexual Health throughout Life

This chapter reviews some issues in the sexual health of Canadian women in adolescence and later life. The focus is on behaviour, knowledge and needs, and includes a review of available indicators of their sexual health. The interpretation and limitations of these indicators are also discussed and policy issues addressed where specific questions need to be answered. Related topics, including sexually transmitted infections, maternal health and contraception, are covered in greater detail elsewhere.

## Methods

### Data

National data on the sexual health of Canadians are scarce, and options for trend analyses or international comparisons are limited. The most recent national data on the sexual health and behaviour of Canadians is the optional sexual behaviour content from the first cycle (1.1) of the Canadian Community Health Survey (CCHS) 2000–2001. The CCHS is a repeated, cross-sectional household-level survey that effectively replaces the cross-sectional component of the National Population Health Survey (NPHS). For more details on the methods and content of the CCHS and NPHS, readers are referred to the Statistics Canada Web site 

Cross-sectional data from cycle 1.1 of the CCHS were analyzed. The data for this report were obtained from individuals aged 15 to 59 at the time of data collection in 2000–2001 and were weighted to represent respondent contributions to the total population, according to CCHS integrated weighting strategy[3].

### Measures

The optional questionnaire module on Sexual Behaviour was used in 57 of 136 health regions nationwide, for a total sample of 55,566. This module was offered only to respondents aged 15 to 59; therefore, the sexual behaviour measures are not nationally representative, but they do capture a large sample of Canadians aged 15 to 59. The content of this module is available to interested readers at the Statistics Canada Web site 

## Results

### Sexual Experience

In weighted analysis, 91.1% of female respondents aged 15 to 59 indicated that they had previously had sexual intercourse. Among males, 89.5% answered that they had previously had sexual intercourse.

### Age at Sexual Debut

Age at sexual debut is typically measured as age at first intercourse. In CCHS 1.1, "sexual intercourse" was not defined for respondents, and this is a common issue in behavioural surveys of sexual activity. Age at first intercourse is important in health terms, as it indicates that an individual may potentially experience pregnancy or a sexually transmitted infection. At the population level, a younger average age at first intercourse results in more sexually active teens and a longer period of sexual activity before a lasting relationship is entered into[4]. However, measuring the first incidence of intercourse unduly suggests that an individual's sexual life commences when they start having penetrative sex. This implied or practical definition of sex also limits our understanding of the sexual activity of gay, lesbian or transgendered women.

**Figure 1:**
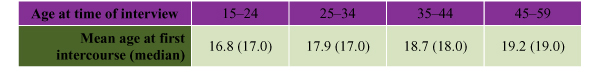
**Age at First Intercourse by Age Group. **Source: Canadian Community Health Survey, Statistics Canada, 2000–2001

The average (mean) age of first intercourse among Canadians responding to the CCHS module on sexual behaviour was 18.4 years. Male respondents were significantly younger than females (mean 18.0 versus 18.8 years respectively, *p *< 0.001). However, the gap narrowed substantially when respondents' age at first intercourse was evaluated by their age cohort. In the youngest age grouping (those aged 15 to 24 at the time of interview), the mean age at first intercourse was 16.7 among males and 16.8 among females (Figure 1).

There was a weak but significant correlation between age of respondent and age at first intercourse (Spearman's *r *= 0.26, *p *< 0.001). This lends support to Maticka-Tyndale and co-authors' findings[4] from the 1996 National Population Health Survey that the median age at first intercourse has steadily declined among younger Canadians. In a comparative study of sexual activity in five developed countries (Canada, United States, France, Great Britain and Sweden) it was found that the age of sexual debut is virtually the same across all five countries[5].

### Differences in Age at First Intercourse

Population health surveys afford little opportunity to evaluate the underlying determinants of sexual health, but some intriguing differences in the initiation of sexual activity are apparent among Canadians.

Respondents to the CCHS module on sexual behaviour who were Canadian-born were significantly younger at first intercourse than their peers who were born elsewhere (mean 17.8 versus 19.9 years respectively, *p *< 0.001). Canadians who define their racial/cultural origin as "white" were similarly younger at first intercourse than those who had other racial/cultural backgrounds (mean 17.9 versus 20.3 years respectively, *p *< 0.001).

Among both males and females, those who had less than a high school education were significantly younger at first intercourse than those who had a high school diploma or any post-secondary education or training (overall mean 17.2 years versus 18.6 respectively). The effect of current household income adequacy on age at first intercourse is negligible overall, but it appears to be more dramatic among women. For female respondents in the bottom two quintiles of income adequacy, the mean age at first intercourse was 17.4 years, whereas among women in the middle to high income quintiles the mean age at first intercourse was 18.9 years (*p *< 0.001). As Maticka-Tyndale et al. note in their similar analysis of 1996 NPHS data,[4] current household income and education may not be the same as that at the time of first intercourse, so any causal relation between these variables cannot necessarily be inferred.

### Sexual Risk Behaviour

The 1998 edition of the *Canadian STD Guidelines *indicates that individuals who have had, among other factors, more than two sexual partners in the previous 12 months and who do not use a barrier method of contraception are at increased risk of sexually transmitted infections (STIs)[6]. This measure of "risk" is an arbitrary one and intended for rapid assessment of sexual history in a clinical setting. Behavioural surveys are a vital means of understanding the characteristics of populations at elevated risk of STIs, with a view to interpreting trends in reported STIs and in planning and evaluating prevention efforts.

Among respondents to the sexual behaviour module in the CCHS 1.1, the vast majority (89.3%) had had sexual intercourse in the previous 12 months, and most of these (90.6%) reported having had only one partner. The proportion of males reporting two or more partners in the previous 12 months was almost double that of females (11.9% versus 6.0%; *p *< 0.001). However, little difference was evident in the proportion of male versus female respondents who consistently used barrier methods in high-risk relationships. Among all those who had one or more relationships lasting less than 12 months in the previous year, only about half (51.6%) reported that they "always" used a condom; 46.9% of the same group indicated that they had used a condom the last time they had had sexual intercourse.

## Discussion

The sexual behaviour module of the CCHS offers a valuable insight into the current sexual behaviour of Canadians. Sexual activity differs in important respects between men and women, and between Canadians of different backgrounds and socio-economic status. However, as is apparent in the results presented here, these measures of behaviour are only a small part of the entirety of sexual health and only apply to a limited population, in terms of both age and geographic region.

Cycle 2.2 of the CCHS is currently in the field. This cycle will include expanded common content on sexual health, which will be directed to all Canadians aged 15 to 49 years. Important questions in this cycle on sexual orientation, history of STIs and contraceptive use will be invaluable in better assessing the sexual health of Canadians. National data currently available do not allow for any assessment of the sexual health or behaviour of lesbian, bisexual or transgendered women in Canada.

The results of the Canadian Youth, Sexual Health and HIV/AIDS survey are in preparation at the time of this chapter's writing. This is the first national survey of the sexual health knowledge and behaviour of school-aged youth since the 1988 Canadian Youth and AIDS survey. Additional instruments, including national sentinel behavioural surveillance of high-risk populations, are currently in development. The status of sexual health measures in Canada indicates that progress is being made in expanding the scope and quality of information available in order to direct resources and shape policy on sexual health in Canada.

Other sources of information on the sexual health of Canadian women have illuminated other aspects of sexual health as well as pointing the way towards important deficiencies in our understanding of sexual health.

### The Foundation of Sexual Health

The physical and social context for lifelong sexual health is established in childhood with appropriate nurturing, stimulation, socialization and education. This foundation enables children to develop a positive sense of self, allowing them to make important life decisions and to cope with life's challenges. It includes the development of trust and intimacy, which in turn contribute to healthy and satisfying relationships[2]. Much of the published research on children and sexual health has focused on childhood sexual abuse rather than on the lifelong benefits of healthy sexual development, a gap that translates into lack of programs and policies in this area.

Gender identity is an important milestone in children's sexual development and is firmly established in most children by the age of 4[7]. Gender roles are first learned in the home, where parents exert the strongest influence, but are subsequently supported by peers and other influences, such as television and school experiences[8]. The attitudes and beliefs that children acquire about gender roles affect their expectations and behaviour, including sexual behaviour, throughout life. However, there are few health promotion initiatives to support the acquisition of healthy and non-biased gender role concepts in childhood.

### Adolescence

#### Definition

Because the adolescent period is typically the beginning of sexual activity, it has received the most attention from researchers in sexual health. However, there is little consensus on what defines adolescent sexuality, and some confusion exists about the concepts of sexuality versus sexual activity.

In Canada, national rates of teenage pregnancy and STI are often used as indicators of the sexual health of adolescents in Canada and for international comparisons[4]. However, these indices represent only a fraction of the relevant issues in adolescent sexuality and not a broad-based behavioural, biological and cognitive approach to adolescent sexual health. One study[9] defined healthy adolescent female sexual development in the following terms:

• The adolescent female experiences positive feelings about her body and its physical changes.

• She recognizes and feels positively about her sexual feelings.

• She involves herself in relationships in which the parties involved take responsibility for their behaviours and consider each person's feelings, needs and desires.

#### Sexual Health Knowledge

Knowledge about sexual health is an important indicator in the development of healthy adolescent sexuality, but knowledge does not necessarily translate into behaviour. Rather, it is necessary to, but not sufficient for, the healthy sexual behaviour of adolescents[10]. For example, teens may know that condoms will help to protect them against unintended pregnancies and STIs but be afraid to suggest their use with partners because they feel this shows a lack of trust. Even if they begin a discussion of condom use with their partner, they may be unable to successfully negotiate the use of condoms. There may be reluctance to purchase condoms because of embarrassment or fear of someone finding out[9].

Langille et al[10]. questioned students 14 to 19 years of age at a Nova Scotia high school on sexual health knowledge, attitudes and behaviour. The results showed that participating adolescents had a high level of knowledge regarding contraception and HIV/AIDS but lacked knowledge in other important areas of sexual health. Students overestimated the sexual activity of their peers, were not always aware of the most fertile periods of a woman's cycle, and 17% believed it was not possible to become pregnant at first intercourse. Despite their reasonable knowledge of HIV/AIDS, their knowledge of chlamydia, another STI, was poor. Fewer than half knew that chlamydia may be asymptomatic, and just over one quarter recognized that chlamydia is common in adolescents. This inequality of sexual health knowledge concerning HIV/AIDS versus other STIs is of concern, given that adolescents are much more likely to become infected with an STI other than HIV[11]. This also suggests that young women may be insufficiently aware of the threat that bacterial STIs pose to their long-term sexual and reproductive health.

Langille et al. noted that, with the exception of the specifics of condom use, females were more knowledgeable than males. In particular, females using oral contraceptives and females with a later sexual debut had higher knowledge scores. However, nearly half of the females surveyed were not aware that parental permission is not required to obtain prescribed oral contraceptives[10].

A somewhat similar survey was administered to inner-city high school students in Toronto by Dell et al. focusing on human papillomavirus (HPV) and other sexual health knowledge. Although HPV is estimated to be the most common sexually transmitted infection in North America, the vast majority of the students surveyed (87%) had not heard of HPV. Understandably females had a better knowledge of Papinocolaou testing than males, but only 39% of sexually experienced females knew who should undergo testing. Once again, both males and females exhibited a greater knowledge of HIV than other STIs and tended to overestimate the prevalence of HIV and underestimate the prevalence of more common STIs such as HPV and chlamydia[29].

The results of these studies are consistent with findings from other countries. Australian and Norwegian adolescents also exhibited a lack of knowledge about chlamydia. Female adolescents in California, as in Nova Scotia, were more knowledgeable than their male counterparts[10]. Similarly, a British study found that higher levels of sexual knowledge were associated with a later onset of sexual activity[12]. This association, perhaps more than any other, underlines the importance of a comprehensive policy on sexual health education and refutes the central tenets of abstinence-based sexual health curricula. Informed and responsible choices about starting sexual relationships and using appropriate protection are best supported when children and adolescents are given access to complete and accurate information.

#### Sexual Attitudes and Communication

Over the last several decades, Canadians have become increasingly comfortable with issues of sexuality and sexual behaviour, and recognize the importance of adolescent sexual health education and access to services[13].

One of the main sources of information on sexual health issues for Canadian youth is family[13]. In one large-scale Canadian study, 59% of adolescent females indicated that they could talk to their mothers and 26% could talk to their fathers about sex; 38% of adolescent males felt they could discuss sexual matters with their mothers and 41% felt they could do so with their fathers[14]. In the 1988 *Canada Youth and AIDS *study, adolescents were asked about their current and preferred sources of information on sex and birth control. Family and school were consistently the top two choices across a range of age groups. Older teens also cited friends as an increasingly popular source of information regarding sex[15].

The ability to talk about sex with potential and current partners is important for adolescents. The *Canada Youth and AIDS *study reported that 81% of school youth indicated they would talk to a sex partner about using a condom[15]. Whether this percentage is as high in actual practice is difficult to assess. Although talking to a partner about using condoms is an important first step, it does not necessarily translate into consistent condom use.

#### Sexual Risk and Protective Behaviours

The context in which adolescent sexual behaviour takes place is often different for males and females. Males are more likely to report that their sexual relationships are "casual" and are more likely to anticipate the possibility of sex with a partner in advance. Adolescent males are less likely to report that they feel remorse or guilt with respect to their sexual activity and experiences[11]. The sexual behaviour of adolescents is complex, being influenced by hormones, desire, peer pressure and the need for acceptance, family and personal values, the media, and other factors[12].

Having had more than one sexual partner in the previous year is considered high-risk behaviour,[5] and in general it is a behaviour that decreases with age[13]. Research indicates that contemporary adolescents and young adults have had more casual and short-term partners than was once the case, resulting in an increased number of sexual partners overall[13]. Adolescent males in Canada (as well as France and Great Britain) are more likely than females to have had multiple partners in the previous year: 38.1% of sexually active Canadian males 18 to 19 years of age versus 23.5% of Canadian females 18 to 19 years, according to one study[5]. The corresponding numbers for both sexes in France were significantly lower but in the United States and Sweden were significantly higher[5].

Condom and contraceptive use is important protective behaviour for sexually active adolescents. In the CCHS 1.1, 49.9% of teens aged 15 to 19 who had had one or more short-term sexual relationships in the previous year reported that they "always" used a condom[3]. One estimate of the rate of *non*-use of any kind of contraception by sexually active Canadian adolescents aged 15 to 19 was 15% among females and 9% among males[4]. The rates of non-use of any contraceptive method at last intercourse were found to be 20% in the United States, 4% to 7% in Sweden and Great Britain, and 12% in France[5].

Some adolescents may not have the maturity to comprehend the long-term consequences of their actions and may be unable to appreciate that having unprotected sexual intercourse now could seriously affect their lives in the future[16]. Unintended pregnancy is one possible consequence of unprotected sex. Although there has been an overall decline in the teenage pregnancy rate in Canada, in 1997 an estimated 19,724 women aged 15 to 19 gave birth, and a slightly larger number in this age range (21,233) had a termination of pregnancy[17].

Canada's teenage pregnancy rate is ranked as moderate compared with other Western industrialized countries[17]. One study reports Canada's rate of adolescent childbearing (the proportion of women aged 20 to 24 who had had a child before age 20) as 11%, as compared with the United States at 22%, Great Britain at 15%, France at 6% and Sweden at 4%[5]. The same study reported the rate of live births per 1,000 women aged 15 to 19 in Canada to be 24.5 (in 1995), as compared with a low of 7.8 in Sweden (1996 data) and a high of 54.4 in the United States (1996 data). Women 15 to 19 in the United States experienced the highest rate of pregnancy overall (83.6/1,000), and Swedish teens the lowest (25.0/1,000). The abortion ratio (number of abortions per 100 pregnancies) was the converse: highest in Sweden and lowest in the United States[5]. Obviously, pregnancy affects the lives of females much more than the lives of males, having a significant, long-term impact on health (for both mothers and babies), socio-economic status and employment prospects.

STIs are another negative consequence of unprotected sex in teenagers, who are disproportionately affected by these infections. This is particularly true of chlamydia, of which the rate in Canada is highest among those aged 15 to 24. Young women may be even more at risk than men because of their physiological differences, which increase their susceptibility to some STIs. Canadian women between the ages of 15 and 24 have a reported chlamydia rate that is nine times the national average[18]. Chlamydia and other STIs can have serious long-term consequences for females, such as pelvic inflammatory disease, ectopic pregnancy and infertility[4]. A more detailed review of STIs and the limitations of surveillance data is presented in another chapter.

### Sexual Health in Later Life

For most men and women, changes in sexual function occur around mid-life and into subsequent decades as a normal consequence of aging. Health care providers and sexually active adults should recognize that as long as individuals maintain overall health, normal aging will not compromise sexual functioning[19].

One of the consequences of the medicalization of sexual health among older adults is that the pharmaceutical industry is a prominent source for information about sexual activity among older Canadians. Despite the focus on intercourse, a survey conducted by Pfizer Inc., manufacturers of Viagra™, indicates that interest in sexual expression does not necessarily diminish with aging. The results of a global study of sexual attitudes and behaviours showed that of 1,000 Canadians between the age of 40 and 80, 73% were sexually active (defined as having had intercourse in the previous 12 months), and most of these individuals (68%) indicated that they had sex more than once per week. The vast majority, 94%, expressed emotional satisfaction, and 91% expressed physical satisfaction with their relationships. Not surprisingly, higher emotional and sexual satisfaction was positively associated with overall health. Canadians were the least likely of any of the 28 nations participating in the global survey to agree with the statement that "older people no longer want sex."[20] Sexual activity in this survey and in much of the literature on the topic is defined exclusively as intercourse, so it is not unreasonable to suppose that many estimates would be even higher if non-coital interaction, fantasy and masturbation were considered within the scope of being "sexually active."[21]

Three basic age-related changes in women's sexual function have been described in both medical and psychological literature. These include diminished desire, diminished sexual responsiveness and dyspareunia (recurrent genital pain associated with sexual activity)[22,23]. Although clinical diagnoses of sexual dysfunction are more common among women than men, at present no treatments are offered specifically for the sexual problems of older women[24]. Population-based rather than patient-based studies more clearly reveal sex differences: older men are more likely to report that they have stopped having sexual relations because they cannot perform sexually, but women are more likely to report that cessation of sexual activity was due to loss or illness of their partner, or the partner's inability to perform sexually[25].

For both men and women, testosterone levels begin to decline in the fifth decade, often resulting in a diminishment of sexual desire[26]. Loss of desire is one of the most commonly presented issues in female sexuality and is particularly difficult to treat, because "desire" is both a psychological and a physical phenomenon[22]. Loss of desire is associated with, and often a consequence of, diminished sexual responsiveness. Estrogen depletion following menopause affects estrogen-sensitive cells throughout the nervous system. Without adequate levels of circulating estrogen, both the psychology and the physiology of sexual response may be affected. Blood flow to vaginal and genital tissues and sensory stimulation are directly affected by declining estrogen levels[27]. These changes can produce vaginal dryness and recurrent pain with intercourse. Dyspareunia is one of the major indications for hormone replacement therapy, but other options, including simply using supplementary lubricant or extending foreplay, may be sufficient for some women[19].

Psychological, cultural and personal factors as well as biological changes contribute to changes in sexual functioning with age. Understanding normal age-related changes is important; however, the medical model of sexuality tends to emphasize the mechanical and negative aspects and give insufficient attention to the underlying feelings associated with sexuality.

Women are perhaps particularly likely to change their sexual self-image through the life cycle. Sexuality takes a more primary role with younger adult women and tends to be associated with fertility and physical beauty. As women age, not only are they potentially less likely to think of themselves as conventionally sexually attractive but they are also increasingly likely to take on more social roles that are higher in priority than the sexual role. When a woman becomes a mother, a professional, a friend, a homemaker, the role of and time allotted to being a lover shrinks[22]. As well, changes in self-image are tied up with notions that mothers/grandmothers/ladies of a certain age are not sexual beings[28].

## Conclusions

### Data Limitations

Sexual development in childhood is an area in which there are significant research gaps. The international study *Health Behaviours of School-Aged Children*, for example, in which Canada is currently participating, surveys Canadian children in grades 6 to 10. However, Canada did not include any content on sexual behaviour in the 1997–1998 questionnaire and, in 2001–2002, questions on sexual behaviour were only asked of respondents in grades 9 and 10. There is considerable reluctance to address questions of sexual knowledge or attitudes to younger children because of concerns about the reliability of responses as well as the need to obtain parental or school board approval. Parents, teachers and health care professionals may feel embarrassed or uncomfortable addressing childhood sexuality; others may underestimate the ability of pre-adolescents to understand and benefit from the promotion of healthy sexuality in children.

Although adolescence has been extensively researched in terms of unintended pregnancies, contraception use and STI, sexual behaviour and sexual knowledge have not been given the same degree of attention. In adulthood, many of the resources directed towards sexual health focus on physiologic sexual dysfunction and not on the psychological aspects of aging and sexuality. The whole spectrum of sexual expression, from masturbation, fantasy, and gender identity to same-sex relationships, has not been comprehensively approached in research or surveillance instruments. Sexual health continues to be perceived as a biomedical construct rather than an integral aspect of total well-being.

### Policy Implications and Recommendations

In all age groups, there appears to be a tendency to focus on the negative aspects of sexuality, such as STIs or unwanted pregnancy. Although they are important, this should not prevent exploration and support of the many positive aspects of sexuality at all ages. Indicators of key differences in sexual behaviour among Canadians suggest that a "one-size-fits-all" approach to sexual health education and promotion is inappropriate and unlikely to be successful.

Sexual health care, education and knowledge are important not only for women of reproductive age but also for all Canadians at all stages of life. This concept needs to be considered when allocating resources and planning future research and programming.

In attempting to determine key indicators and comparisons, it becomes apparent that consistent measures of the sexual health of Canadians have been sorely deficient and only now is the deficit beginning to be remedied. In keeping with a broad and lifelong approach to sexual health, our recommendations include a greater emphasis on the continuum of sexual activity and expression. Rather than limiting investigations to intercourse and its sequelae, approaching sexual health in the context of relationships, communication strategies, and attitudes and knowledge would foster a deeper understanding of what constitutes healthy sexuality and the structural or policy changes needed to promote it.

## Notes

The views expressed in this report do not necessarily represent the views of the Canadian Population Health Initiative, the Canadian Institute for Health Information or Health Canada.

The authors would like to acknowledge the contribution of Mike Barrett PhD.
